# Physicochemical Characterization, Storage Stability Behavior, and Intestinal Bioaccessibility of Clove Extract Encapsulated Using Varying Combinations of Gum Arabic and Maltodextrin

**DOI:** 10.3390/foods14020237

**Published:** 2025-01-14

**Authors:** Farhad Ahmadi, Hafiz A. R. Suleria, Frank R. Dunshea

**Affiliations:** 1 School of Agriculture, Food and Ecosystem Sciences, Faculty of Science, The University of Melbourne, Parkville, VIC 3010, Australia; hafiz.suleria@unimelb.edu.au (H.A.R.S.); fdunshea@unimelb.edu.au (F.R.D.); 2 Faculty of Biological Sciences, The University of Leeds, Leeds LS2 9JT, UK

**Keywords:** antioxidant activity, bioactive compound, clove extract, polyphenol, wall material

## Abstract

Clove (*Syzygium aromaticum*, L.) is a rich source of polyphenols and antioxidants, but its intense flavor, poor solubility, and instability may limit its widespread and efficient use in industrial applications. In a series of laboratory-scale experiments, gum Arabic (GA) and maltodextrin (MD) were used as coating agents in various proportions (ranging from 0MD:100GA to 100MD:0GA) for encapsulation of clove extract using a freeze-drying method. The encapsulates were assessed for the physicochemical properties, storage stability behavior, and intestinal bioaccessibility of phenolics using an *in vitro* gastrointestinal digestion test. The freeze-dried encapsulates were characterized as having low water activity (<0.3, which is a critical threshold to ensure chemical and microbiological stability), high water solubility (>90%), solid (product) recovery (mean 93.1 ± 1.77%), and encapsulation efficiency (91.4−94.9%). Hygroscopicity increased as the GA:MD proportion increased in the encapsulation formulations. Encapsulation was effective in protecting bioactive components of clove extract during storage at room (up to 40 days) or high temperature (60 °C for 7 days) and minimized the loss of antioxidant activity during storage, as compared to the clove extract in a non-encapsulated form. All encapsulation formulations were characterized by a negative zeta potential (from −22.1 to −29.7 mV) and a polydispersity index ranging from 0.47 to 0.68, classifying the formulations as having a mid-range polydisperse particle size distribution. The FTIR analysis demonstrated that the freeze-drying encapsulation process resulted in no evident chemical interaction between coating and core materials. Intestinal bioaccessibility of total phenolics after the *in vitro*-simulated gastrointestinal digestion was greater in the encapsulated clove extract compared to the non-encapsulated clove extract. In conclusion, the encapsulation process was effective in protecting the bioactivity of the polyphenol-rich clove extract during storage and improved the phenolic bioaccessibility, potentially supporting the application of the encapsulated clove extract for use in functional food development.

## 1. Introduction

Phytochemicals, secondary metabolites in plants, have been demonstrated to have various beneficial properties, including growth enhancement, antimicrobial effects, antioxidant capabilities, anti-inflammatory benefits, and immunostimulant activities [1]. Clove has demonstrated a wide range of biological activities, attributed primarily to its rich polyphenol content [2], most importantly eugenol, its major bioactive compound. Eugenol is generally recognized by FDA classification as safe for use as a food additive, which has contributed to its growing interest and application within the food and pharmaceutical industries [3,4]. However, the intense odor, bitter flavor, poor solubility, and volatility and instability when exposed to high temperature, low pH, or oxygen have limited its widespread use in industrial applications [5,6].

To ensure commercial viability, plant bioactives should maintain their quality regarding bioactivity and safety for animal or human consumption during prolonged storage across varying conditions. Encapsulation has emerged as a promising technique enabling sensitive bioactive components to be enclosed within an encapsule matrix, thereby protecting them from unfavorable external conditions [7,8]. Encapsulation can help to maintain functionality and bioactivity of encapsulates during storage, as well as to mask undesirable tastes and control the release of the core active components during gastrointestinal digestion [6,9]. Importantly, choosing wall materials for encapsulation is crucial to achieving these benefits.

Gum Arabic (GA) and maltodextrin (MD) were selected for this purpose because of their widespread availability, high solubility, safety, and frequent use in food product development [10,11]. For example, MD is a popular carrier agent for encapsulation because of its high water solubility, low viscosity, free-flowing properties, flavor-blending capability, effective binding properties, and its capacity to protect bioactives from oxidation [12]. Past studies suggest that the stability and encapsulation efficiency of encapsulates made with MD can be further improved by incorporating GA, a long-chain polysaccharide offering high solubility, effective emulsifying properties, and low viscosity in aqueous solutions [13,14].

This study investigated the feasibility of developing an encapsulated clove extract product using various proportions of MD and GA. The primary objectives were to determine the optimal proportion of MD and GA to achieve the most efficient encapsulated powders with superior technological properties, maintain the storage stability of clove extract polyphenols, and increase their bioaccessibility at the intestinal level.

## 2. Materials and Methods

### 2.1. Chemical and Reagents

Folin–Ciocalteu reagent, gallic acid, ascorbic acid, sodium phosphate, iron chloride hexahydrate, sodium carbonate, 2,4,6-tripyridyl-s-triazine (TPTZ), and eugenol (99% purity; E51791) were purchased from Sigma-Aldrich (Castle Hill, NSW, Australia). Methanol, ethanol, glacial acetic acid, and formic acid were purchased from Thermo Fisher Scientific Inc. (Scoresby, Victoria, Australia). Milli-Q water was collected using a Millipore Milli-Q gradient water purification system (Darmstadt, Germany). Pancreatin was acquired from Southern Biological (Melbourne, Victoria, Australia), and α-amylase from *Aspergillus oryzae* was purchased from Sigma-Aldrich (Castle Hill, NSW, Australia). Gum Arabic from the acacia tree and maltodextrin (dextrose equivalent 16.5–19.5) were purchased from Sigma-Aldrich (Castle Hill, NSW, Australia).

### 2.2. Clove Extract Preparation

A clove bud was purchased from a local market (Mudbrick Herb Cottage, Queensland, Australia) and ground into a fine powder form using a grinder (Cuisinart^®^ Spice and Nut Grinder, SG-10A, Asquith, NSW, Australia). A three-step extraction process was initiated with 80% methanol at a sample-to-solvent ratio of 1:10 and shaken at 150 rpm in 4 °C for 2 h in a Labwit ZWYR-240 incubator shaker (Ashwood, Victoria, Australia). Then, this slurry was centrifuged (Hettick ROTINA380R Refrigerated Centrifuge; Tuttlingen, Baden-Württemberg, Germany) at 8000× *g* for 10 min at 4 °C. The supernatant was harvested and filtered through a Whatman No. 1 filter paper. This extraction process was repeated twice with the shaking and centrifugation specifications described above. All supernatants collected were pooled, mixed, and concentrated using a BÜCHI rotary evaporator at 40 °C under reduced pressure until about 95% of the solvent was evaporated. The concentrated extract was stored at −80 °C and subsequently freeze-dried using a Biobase Benchtop freeze dryer (BK-FD10S, Biobase, Jinan, China) for 72 h. The freeze-dried extract powder was used in the encapsulation process.

### 2.3. Encapsulation by Freeze-Drying Method

Maltodextrin and GA were used as encapsulating agents in various proportions, as listed in Table 1. The encapsulation process was performed following the procedure of Parvez et al. [12], with some modifications. In brief, distilled water was added to the wall materials to create a 30% *w*/*v* solution, then left overnight (4 °C) to ensure complete saturation of the polymer. Freeze-dried clove extract was then mixed with the wall material solution at a 1:4 ratio. This mixture was homogenized using a high-speed homogenizer (Ultra-Turrax^®^ T25; IKA Inc., Wilmington, NC, USA) at 9500 rpm for 15 min at room temperature [15]. The resulting solution was frozen at −80 °C for 24 h and then freeze-dried for about 72 h. After freeze drying, the encapsulated biomass was ground with a pestle and mortar [16]. The encapsulated powders were kept in an airtight amber bottle under refrigeration.

### 2.4. Total Phenolics

The total phenolic content was quantified using the Folin–Ciocalteu procedure [17]. A diluted Folin–Ciocalteu reagent (1:3; 25 μL) was added to the sample extract (25 μL), derived either from the encapsulated powder or the clove extract powder in non-encapsulated form, followed by adding 200 μL of distilled water. A total of 25 μL of 10% (*w*/*w*) sodium carbonate was introduced after 5 min incubation. The mixture was then incubated for 60 min at room temperature, and absorbance was read at 765 nm using a Multiskan^®^ Go Microplate spectrophotometer (Thermo Fisher Scientific, Waltham, MA, USA). A standard curve of gallic acid (0−200 μg/mL) was created and used to express the total phenolic content as mg gallic acid equivalent (GAE)/g dry matter.

### 2.5. Ferric Reducing Antioxidant Power (FRAP) Assay

A FRAP assay was undertaken following the modified protocol of Wang et al. [18]. The dye solution for this assay was created by combining 300 mM sodium acetate, 10 mM TPTZ, and 20 mM Fe [III] solutions (10:1:1, *v*/*v*/*v*). The dye solution (280 μL) was added to 20 μL of the sample extract derived either from the encapsulated or non-encapsulated clove extract and incubated for 10 min. A standard curve was created using Trolox (0 to 100 μg/mL). Absorbance was read at 593 nm. The values were expressed in mg Trolox equivalent (TE)/g dry matter.

### 2.6. Encapsulation Efficiency

The procedure of Saikia et al. [19] was adopted to determine the encapsulation efficiency. Initially, surface phenolics were extracted by mixing 200 mg of the encapsulated sample with 2 mL of a 1:1 methanol–ethanol solution. The mixture was vortexed for 1 min and then filtered using a 0.45 μm acrodisc syringe filter and subjected to quantification of total phenolic using the Folin–Ciocalteu reagent assay as described in Section 2.4. For the harvest of total phenolics entrapped in the core material, a 200 mg encapsulated sample was dissolved in 2 mL of a solvent system with a volumetric ratio of 50:8:42 (*v*/*v*/*v*) of methanol, acetic acid, and water. The mixture was vortexed for 20 min and then subjected to ultrasonic treatment (John Morris Scientific, Melbourne, VIC, Australia). After centrifugation (10 min at 2057× *g*), the supernatant was collected upon filtration using an acrodisc syringe filter. Total core phenolics were quantified using the Folin–Ciocalteu reagent assay as described in Section 2.4. The encapsulation efficiency was computed using the following Equation:

Encapsulation efficiency (%) = [(TCP − SP)/TCP] × 100(1)

where SP = surface phenolics, and TCP = total core phenolics.

### 2.7. HPLC Quantification of Eugenol

A quantitative analysis of eugenol was performed following an HPLC method developed and validated by Yun et al. [20], with slight modifications. Briefly, liquid chromatography was performed using an Agilent 1200 HPLC system (Agilent Technologies, Santa Clara, CA, USA) attached with a DAD at 280 nm. Eugenol was separated in a Synergi Hydro-RP 80 Å, LC Column (250 mm × 4.6 mm, 4 μm particle size) (Phenomenex, Lane Cove, NSW, Australia). The mobile phase was methanol–water (60 + 40, *v*/*v*). A 20 µL aliquot of each sample extract (from the clove extract in encapsulated or non-encapsulated form) was filtered through a 0.45 µm syringe filter (Thermo Fisher Scientific Inc., Waltham, MA, USA) and set for injection at a flow rate of 0.8 mL/min. The column temperature was maintained at 35 °C. Quantification of eugenol was achieved through a calibration curve construction (Supplementary Figure S1) and peak area measurements.

### 2.8. Physicochemical Characterization

#### 2.8.1. Water Solubility, Water Activity, and Hygroscopicity

Water solubility was determined using the method described by George et al. [15]. Approximately 1 g of the encapsulated powder was mixed with 25 mL of distilled water and mixed vigorously. The mixture was incubated at 35 °C for 30 min and subsequently centrifuged at 4000 rpm for 4 min. The supernatant was transferred to a pre-weighed Petri plate and dried in an oven of 105 °C. Water solubility was calculated as the ratio of the dried mass of supernatant to the initial weight of the powder sample. Water activity was measured at 25 °C using a Novasina LabMaster-aw analyzer (Lanchem, Switzerland).

Hygroscopicity was determined following the procedure of Rezende et al. [16]. A 1 g encapsuled powder was transferred in a desiccator with a saturated solution of sodium chloride (relative humidity = 75.3%). The powders were weighed after 7 days, and the amount of adsorbed moisture was determined.

#### 2.8.2. Flow Properties

Bulk density was assessed using the method outlined by Premi et al. [21]. A 2 g sample of the encapsulated powder was placed into a 10 mL graduated glass cylinder and tapped 10 times from a height of 10 cm. Bulk density was then calculated as the mass of the powder divided by the volume it occupied in the cylinder. Tapped density was determined by manually tapping the cylinder from a 10 cm height until the volume stabilized. The flow properties of encapsulated powders, including Carr index and Hausner’s ratio, were determined using the following Equations [7]:

Carr index = (Tapped density − Bulk density)/Tapped density × 100(2)

Hausner’s ratio = Tapped density/Bulk density(3)

#### 2.8.3. Colorimetric Analysis

Colorimetric analysis of encapsulated powders and clove extract (non-encapsulated) was performed using a portable colorimeter (Chroma Meter CR-400, Konica Minolta, Osak, Japan) using CIELAB color coordinates (*L*, *a*, and *b*), where *L* represents brightness, ranging from 0 (black) to 100 (white), *a* represents the color shift from green (negative) to red (positive), and *b* represents the color shift from blue (negative) to yellow (positive).

#### 2.8.4. Product Recovery and Loading Efficiency

Solid (product) recovery was calculated as the proportion of the encapsulated product recovered after the freeze-drying process relative to the total solids before freeze drying using the following Equation [22]:

Solid recovery (%) = *M*_f_/*M*_t_ × 100 (4)

where *M*_f_ = solid mass of the encapsulated product after freeze drying upon exit from the freeze dryer. *M*_t_ = solid mass before freeze drying (encapsulating agent + active compound)] × 100.

The loading efficiency of total phenolics and eugenol was calculated as the ratio of their concentration in the freeze-dried encapsulates to their theoretical concentration prior to the freeze-drying process, according to the following Equation [23]:

Loading efficiency (%) = *C*_a_/*C*_t_ × 100 (5)

where *C*_a_ = concentration of total phenolics or eugenol in encapsulated samples after freeze drying. *C*_t_ = theoretical concentration of total phenolics or eugenol prior to the freeze-drying process. Moisture content was determined from the weight loss after heating the samples in a drying oven at 105 °C until a constant weight was achieved [24].

#### 2.8.5. Polydispersity Index and Zeta Potential

Polydispersity index (PDI) was determined, in both the encapsulated and non-encapsulated clove extract forms, by using a Zetasizer Nano ZS (Zen 3600, Malvern Instruments, Malvern, UK). The measurements were conducted at 25 °C, with the laser beam set at an incidence angle of 173°. The zeta potential (mV) was measured using the same Zetasizer equipment. Sample preparation involved dispersion of encapsulated powder in Milli-Q water and sonication treatment to achieve an approximate concentration of 10 mg/mL, ensuring that the solution was sufficiently diluted to minimize multiple scattering effects [25]. The readings were performed by transferring 0.8 mL of the emulsion into a DTS1070C polystyrene bucket at 25 °C.

#### 2.8.6. Fourier Transform Infrared (FTIR) Spectroscopy

FTIR analysis was undertaken using an Alpha II compact FTIR spectroscopy (Bruker, Billerica, MA, USA) to detect changes in functional groups within the encapsulated samples. The samples (encapsulated powder or clove extract powder in non-encapsulated form) were prepared by pressing about 5 mg of the material onto a diamond crystal. Spectra were recorded by averaging 16 scans/sample from 4000 to 400 cm^−1^ at a resolution of 4 cm^−1^. Air was used as the background. The scan rate was 32 scans min^−1^. Normalization of FTIR data (to the range 0 to 1) and graphical presentation of spectra were made in Origin Pro (Version 2024b. OriginLab Corporation, Northampton, MA, USA).

### 2.9. Storage Stability Test

The encapsulated powder and the non-encapsulated clove extract were each divided into two allotments of approximately 2 g each, placed in screw-capped tubes, and stored in the dark at either room temperature (25 °C) or cold temperature (4 °C). The storage duration was 40 days, and the sample collection occurred at 10-day intervals. At each interval, the samples were analyzed for surface and core phenolic content, encapsulation efficiency, antioxidant capacity (using FRAP assay), and eugenol concentration.

An accelerated storage stability test was also undertaken to identify which encapsulation formulation offers superior resistance against high temperatures. Approximately 2 g of the encapsulated or non-encapsulated clove extract powder was placed in a sealed tube and stored in an oven at 60 °C for 7 days in the dark. After this incubation period, the total core phenolics (as outlined in Section 2.4), antioxidant activity (using FRAP assay as outlined in Section 2.5), and eugenol concentration (as outlined in Section 2.7) were quantified.

### 2.10. In Vitro Gastrointestinal Digestion

A modified version of the static *in vitro* gastrointestinal digestion method [26] was employed, following the modifications outlined by Grace et al. [27]. In brief, stock electrolyte solutions of simulated salivary, gastric, and intestinal fluids were prepared according to the reference paper [26]. The oral phase was initiated by suspending 100 mg of the clove extract or 200 mg of the encapsulated sample in powder forms with water (1 mL). Then, 0.7 mL of the salivary fluid was added to the mixture, followed by adding 100 µL of 1500 U/mL porcine pancreas α-amylase solution, and then sequentially adding 50 μL of 0.03 M CaCl_2_ and 150 μL of water. The whole mixture was thoroughly mixed for 2 min. The gastric phase was initiated by combining the oral bolus (2 mL) with the gastric fluid (1.28 mL), 25,000 U/mL porcine pepsin stock solution (320 μL), and 0.03 M CaCl_2_ (10 μL). The pH was adjusted to 3.0 using HCl. Water was added to achieve a final volume of 4 mL. The mixture was then incubated at 37 °C in a shaking incubator for 2 h. For the intestinal phase, gastric chyme (4 mL) was combined with the intestinal fluid (2.2 mL), 800 U/mL pancreatin solution (1 mL), fresh bile (160 mM; 0.5 mL), and 0.03 M CaCl_2_ (80 μL). The pH was adjusted to 7.0 with NaOH. Water was added to achieve a final volume of 8 mL. The solution was shaken for 2 h at 37 °C. At the end of the digestion phase, samples were centrifuged to separate the soluble fraction from the residual fraction. The supernatant harvested by centrifugation (8000× *g* for 15 min) was immediately snap-frozen, awaiting analysis to quantify the total phenolic content as described in Section 2.4. The intestinal bioaccessibility (%) of total phenolics in both the non-encapsulated and encapsulated clove extract forms was calculated using the following Equation:

Intestinal bioaccessibility of phenolics = *PH*_f_/*PH*_i_ × 100 (6)

where *PH*_f_ = total phenolic content quantified in intestinal supernatant after the complete digestion process, and *PH*_i_ = total phenolic content quantified in samples prior to *in vitro* digestion [27].

### 2.11. Statistical Analysis

Data analysis was run in SAS (version 9.4, SAS Institute Inc., Cary, NC, USA). Before analysis, the dataset was checked again for potential outliers, and a normality test was performed, but no transformation was needed as the data were normal. The data were collected over 3 separate experimental runs. The run was included in the model as a random variable. The results are presented as mean ± standard deviation. A Tukey’s multiple range test was employed for the means comparison. The significance threshold was set at *p* < 0.05.

## 3. Results and Discussion

### 3.1. Characterization of Clove Extract

The yield of the clove extract powder using methanolic extraction was 15.1 ± 1.04 g extract/100 g dry clove bud, which is comparable to the value of 11.7 g extract/100 g clove bud (using 80% ethanolic extraction) as reported by El-Maati et al. [28]. The total phenolic content of the methanolic clove extract was determined to be 301 ± 7.32 mg GAE/g dry extract, which is comparable to the 352 mg GAE/g extract obtained through ethanolic extraction [29]. However, this value was higher than the 201 mg GAE/g dry clove extract obtained via methanolic extraction reported by Elhussein et al. [30]. In contrast, a much higher phenolic content of 507 mg GAE/g clove extract was reported through the maceration method using 70% ethanol [6]. The type and concentration of solvent used in the extraction process, along with the maturity of the clove bud, are factors that may result in the discrepancies observed in the extraction yield and total phenolic content [6].

The eugenol concentration was 87.7 ± 2.52 mg/g clove extract, exceeding the 26.9 mg/g dry weight quantified in the ethanolic clove extract [31]. Conversely, Johannah et al. [32] reported a lower concentration of 9.4 mg/g clove bud, which can become more comparable to the results of this study when adjusted for an extraction yield of 15.1%. These large variations can be attributed to various factors influencing handling and processing methods for the preparation of herbal products, including environmental conditions, harvest timing, drying methods, storage practices, as well as the extraction procedures [20].

### 3.2. Physicochemical Properties

#### 3.2.1. Moisture Content, Water Solubility, Water Activity, and Hygroscopicity

Physicochemical properties of encapsulated powders are reported in Table 2. The 0MD:100GA formulation had the highest moisture content (5.30%), and as the proportion of MD increased in encapsulation formulation, the moisture content decreased, reaching the lowest value of 1.30% in the 100MD:0GA formulation. Consistent with this result, Kang et al. [33] also reported that as the MD concentration increased in the wall materials, as compared to GA, the moisture content of the encapsulates decreased. This effect was attributed to the higher number of hydrophilic groups in GA, which can bind more water molecules, leading to increased moisture content when GA is present in encapsulates in greater proportion [34]. The moisture content is critical for the storage stability of encapsulates, as a higher moisture level may promote the release and degradation of the core material during storage.

The encapsulated powders developed in this investigation demonstrated a generally high water solubility, with the highest solubility determined in the encapsulates developed solely of MD (water solubility = 95%) and the lowest in the encapsulates developed only of GA (water solubility = 89.9%). This may suggest that the clove extract encapsulated with greater MD proportion can be readily and rapidly reconstituted. This high solubility observed across the formulations can be attributed to the inherently high solubility of both GA and MD [35], comprising 80% of the encapsulated powders. In support of our findings, previous studies [14,36] have also reported the higher solubility of MD over GA. Solubility is a critical property of encapsulated powders, as the release of bioactive compounds entrapped within the wall materials relies on the dissolution of the encapsulating agent. Encapsulation using appropriate wall materials usually enables the transformation of hydrophobic compounds into more hydrophilic forms, allowing them to be incorporated more efficiently into water-based food matrices [37]. In both food and pharmaceutical industries, low solubility is undesirable because it may limit efficient dissolution in food matrices or incorporation into specific formulations, leading to processing difficulties and reduced bioavailability [38,39].

There was no significant difference in water activity across the encapsulates coated with different ratios of GA and MD (mean = 0.26 ± 0.03; *p* = 0.17). This value falls within the water activity values typically observed in industrially developed encapsulates via the freeze-drying method. Importantly, this average water activity falls within the recommended threshold of less than 0.3, which is crucial for ensuring microbiological stability, preventing stickiness, and reducing the risk of agglomerate formation during long-term storage [38,40]. Water activity, a measure of equilibrium moisture in a biomass, is defined as the point where a material no longer releases or absorbs water into its surroundings. This physical property is a critical factor in preventing the growth and proliferation of microorganisms, tending to thrive at higher water activity contents. Similarly, Laureanti et al. [38] found no significant differences in the water activity of pink pepper encapsulated using a combination of GA and MD with the freeze-drying method.

As the GA:MD proportion increased in the formulations, the hygroscopicity of the encapsulates increased, which is consistent with the findings of Silva et al. [41], reporting that the encapsulates produced with a higher MD proportion were less hygroscopic. Lower hygroscopicity is a desired property as it indicates that the encapsulated powders absorb less moisture from the surrounding environment, which is important from the viewpoint of maintaining the physicochemical and storage stability and flowability properties [14]. Generally, the hygroscopicity of the encapsulated powders is influenced by factors such as the chemical structure, type, and concentration of the carrier agents; the encapsulation method (freeze drying or spray drying); the size of encapsulated powders; and the moisture content [21,42]. For example, a direct association between the moisture content and hygroscopicity has been reported [21], which was also demonstrated in this investigation. As reported in Table 2, the highest numerical hygroscopicity (10.1%) was recorded in the 0MD:100GA formulation, which was also characterized by the highest moisture content (5.30%), indicating the higher water retention capacity of GA.

#### 3.2.2. Flow Properties

Data on the flowability properties are listed in Table 2. The highest bulk and tapped densities were recorded in the 100MD:0GA formulation, averaging 0.353 and 0.439 g/cm^3^, respectively. Generally, as the GA proportion increased in the formulation of encapsulates, the bulk and tapped density tended to decrease. This observation is consistent with the findings of Zhang et al. [43], reporting that encapsulated powders produced with GA had lower bulk and tapped density than those produced with MD. The lower bulk density of encapsulates developed using a higher GA proportion has also been ascribed to the viscous nature of GA [15]. A higher bulk density is preferable, as it allows for more efficient storage, occupying less space during packaging and transportation [15]. Overall, regardless of the coating agent combinations, the encapsulated powders were characterized by low density. This is a common characteristic of encapsulated powders produced using the freeze-drying method, mainly because of their irregular morphological structure, forming hollow areas between the particles [7,44].

Table 2 lists data on the flowability and cohesiveness of the encapsulated powders, represented by the Carr index and Hausner’s ratio, respectively. Carr index and Hausner’s ratio showed no significant variation (*p* > 0.05) across the encapsulated powders, with average values of 23.3% (19.7–24.7) and 1.31 (1.25–1.33), respectively. The Carr index values across different formulations fell within the fair flowability range (20–35%) according to the benchmark classifications [45]. The amorphous structure of the encapsulated powders and their low moisture content (Table 2) may have influenced the cohesive and frictional forces, affecting the flowability properties [44]. In addition, the small particle sizes and large surface area of the encapsulated powder are known to result in increased cohesive and frictional forces that may resist flow [46].

#### 3.2.3. Colorimetric Analysis

Color characterization of freeze-dried encapsulated powders, as listed in Table 3, showed a distinct relationship between the composition of MD and GA and the lightness of the encapsulated powders. The lowest *L* value was observed in the 0MD:100GA formulation, while the highest *L* value was recorded in the 100MD:0GA formulation. There was no difference in the *L* value of the formulations containing the varying blends of MD and GA. The highest *a* value belonged to clove extract (average = 14.8), reflecting its deep brown color. However, after its incorporation into the wall materials, there was no significant difference in the *a* value of the encapsulated powders, which averaged 11.2 ± 0.37. The obvious difference in *L* value between the 100MD:0GA and 0MD:100GA formulation is likely attributed to the inherent color properties of GA, and MD:GA typically exhibits a yellow to a reddish hue, which lowers the *L* value, whereas MD is white, leading to an increase in the *L* value as its proportion rises in the formulation [47]. A similar observation was reported by Kang et al. [33], encapsulating chlorophylls using different blends of GA and MD.

### 3.3. Solid Recovery and Loading Efficiency

Data on solid recovery and loading efficiency of total phenolics and eugenol is reported in Table 4. Solid recovery was not different (*p* = 0.84) across the formulations, averaging 93.1 ± 1.77%. Contrary to this high solid recovery using MD and GA observed in this study, Sarabandi et al. [14] utilized GA, MD, or their 1:1 combination for encapsulation of eggplant peel extract through the spray-drying process and reported much lower solid recoveries of 39.6%, 52%, and 47.3%, respectively. This clearly highlights the efficacy of the freeze-drying method (as opposed to the spray-drying method) in achieving a high solid recovery rate when using GA and MD as carrier agents. Consistent with these findings, Estupiñan-Amaya et al. [48] reported a high solid recovery of 94–96% in blueberry encapsulated via the freeze-drying method using various combinations of MD and GA.

The loading efficiency of total phenolics and eugenol was not different (*p* > 0.05) across the encapsulation formulations developed using various combinations of MD and GA. On average, there was a 20.4% loss of phenolics and a 26.6% loss of eugenol across the different formulations (Table 4). Loading efficiency provides an estimation of the proportion of initial bioactives recovered in the final product after freeze-drying process, thereby serving as an indicator of the efficiency of the encapsulation process. Losses may result from the precipitation of less water-soluble compounds likely lost during sample preparation steps such as solubilization, centrifugation, and filtration [23]. Moreover, some phenolic compounds may degrade after grinding freeze-dried products, increasing their exposure to the oxidation reactions [49].

### 3.4. PDI and Zeta Potential

As shown in Table 5, the PDI value for all encapsulates was within the range of 0.47 to 0.68, classifying the encapsulates as mid-range polydispersed particles [50]. PDI is a key metric for assessing the uniformity of particle size distribution in colloidal systems, with values ranging from zero (a monodispersed sample) to 1 (a polydispersed sample). The clove extract in non-encapsulated form had a mean PDI of 0.52 and encapsulation using different wall compositions resulted in no significant effect on PDI value. Numerically, encapsulates developed using a 87.5MD:12.5GA formulation had the lowest PDI (0.47), which may suggest their more uniform particle size distribution in aqueous solution.

A negative zeta potential was determined in all encapsulates, ranging from −22.1 mV in 50MD:50GA to −29.7 mV in 100MD:0GA. The clove extract without encapsulation also had a negative zeta potential (−28.6 mV). Zeta potential is a metric that provides information on the electrostatic repulsive forces between particles suspended in an aqueous medium, reflecting the stability and distribution of microparticles in a liquid environment [51]. A zeta potential value near −30 mV typically signifies the threshold for stability in particle suspension. Emulsions with values lower than −30 mV or greater than +30 mV tend to be electrostatically stable, enabling sufficient repulsive forces to prevent particle aggregation [25]. The data presented in Table 5 suggest that zeta potential measurements for the encapsulated clove extract did not show a significant improvement in stability compared to clove extract in non-encapsulated form. Further investigation is needed to assess if incorporating other wall materials in combination with GA or MD can further improve the stability and distribution of encapsulated clove extract in an aqueous medium.

### 3.5. FTIR Spectroscopy

**Figure 1:**
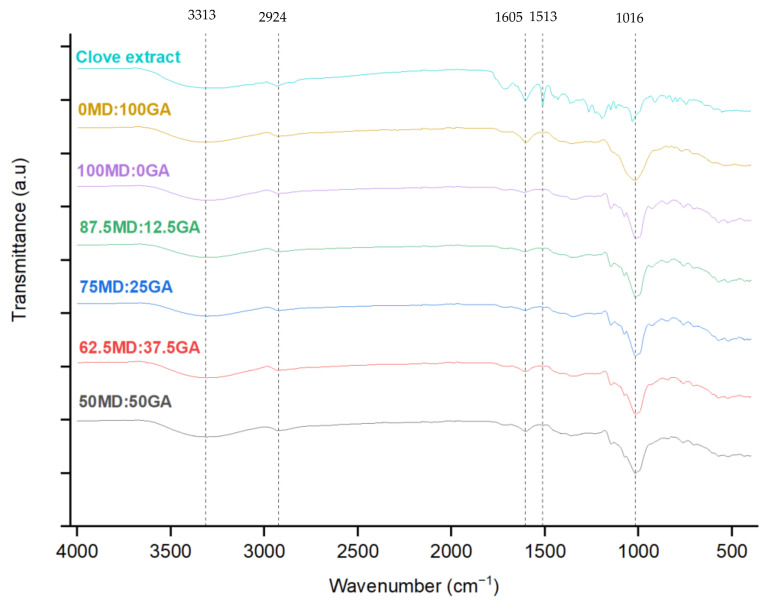
Fourier transform infrared (FTIR) spectra of clove extract in non-encapsulated or encapsulated forms. MD = maltodextrin, GA = gum Arabic. Proportion of MD and GA in the formulations is presented in Table 1.

Figure 1 shows the FTIR spectra in the range of 400–4000 cm^−1^ for the freeze-dried clove extract in both non-encapsulated and encapsulated forms, using the varying blends of MD and GA as wall materials. This analysis was used to identify potential interactions between the coating (MD and GA) and the core materials (clove extract). In non-encapsulated clove extract, a characteristic peak at 1513 cm^−1^ attributed to the asymmetric C=C stretching of the aromatic moiety was identified, which confirms the presence of eugenol, the primary phenolic compound in clove [52,53]. However, in the encapsulated powders, the intensity of this peak was significantly reduced, aligning with a lower eugenol concentration in the encapsulated powders (Table 6). This reduction was expected because of the dilution effect from the encapsulating matrix, where the 1:4 clove extract-to-wall material ratio decreased eugenol content in the final product.

In the 3500–3000 cm^−1^ region, both the non-encapsulated clove extract and the encapsulated powders exhibited hydrogen bonding and –OH groups associated with the phenolic compounds in the extract. Further, this region indicates the stretching vibrations of –OH groups from residual water that remained in the samples after the freeze-drying process [54]. Peaks between 900 cm^−1^ and 1600 cm^−1^ are mainly linked to phenolic compounds, with the 1605 cm^−1^ peak indicating C=C bond vibrations in phenolic and aromatic compounds [55]. The presence of this bond in both the non-encapsulated and encapsulated powders may possibly suggest that the phenolic structures may have been preserved during the encapsulation process.

Overall, the FTIR spectra of the freeze-dried clove extract in non-encapsulated and encapsulated forms were indicative of the formation of no new chemical bonds, likely suggesting that the polyphenol-rich clove extract was encapsulated using the varying ratios of MD and GA without altering its chemical properties while preserving its bioactive compounds [25]. Similar FTIR patterns were reported by Nguyen et al. [25], encapsulating noni fruit extract using GA and MD through the spray-drying method.

### 3.6. Storage Stability Behavior

Data on the surface and core phenolic content, encapsulation efficiency, eugenol concentration, and antioxidant capacity (measured as FRAP) as a function of different proportions of carrier agents, storage temperature, and storage durations are tabulated in Table 6. Surface phenolics were affected by both storage temperature and duration. As the storage duration progressed, surface phenolic concentration increased, the extent of which was slightly greater in the encapsulates kept in room vs. cold temperature. Among the carrier formulations, surface phenolics existed in lower concentrations in the 0MD:100GA formulation. This difference can likely be attributed to the physicochemical properties and interaction mechanisms of MD and GA. The branched chain structure and complex polysaccharide nature of GA [56] likely contribute to the formation of a more intricate and robust matrix, potentially enabling more effective trapping of the phenolic compounds within the core and limiting their diffusion to the surface. In support of this observation, Sid et al. [57] reported that surface phenolics were lower in encapsulated Kinnow peel powder developed with a higher proportion of GA (lower MD:GA proportion).

There was no significant difference (*p* = 0.26) across the coating combinations on total core phenolic content over the 40-day storage duration at both room and cold temperatures. However, irrespective of coating combinations and storage temperature, there was a small loss in total core phenolics after 40 days of storage (7.8%; *p* = 0.04). Consistent with these findings, Vonghirundecha et al. [58] reported that encapsulation of Moringa oleifera leaf extract with MD resulted in a slight loss in total phenolic content and antioxidant capacity at 4 °C vs. 37 °C, over 90 days of storage. Past studies suggest that a higher bulk density may reduce air content in encapsulated powders, and thus minimize air-related degradation processes [15,59]. However, such an effect was not observed in this investigation, as despite the differences in bulk density across the formulations (Table 2), total core phenolics remained unaffected across the encapsulates with the varying wall material composition.

As the GA proportion increased in the formulation, the encapsulation efficiency tended to increase, with the greatest encapsulation efficiency identified in 0MD:100GA formulation (about 95%; Table 6). As the storage duration prolonged, the encapsulation efficiency decreased (*p* < 0.05). However, the storage temperature had no significant effect (*p* = 0.12) on the encapsulation efficiency. Encapsulation efficiency is an indication of the success of the encapsulation process, allowing for assessing the quality of the protection offered to the bioactive components (core component) embedded within the wall materials [60]. Our findings demonstrated the successful achievement of a high encapsulation efficiency (ranging from 91.6% in 100MD:0GA to 95.0% 0MD:100GA in 0-day encapsulates; Table 6) using the freeze-drying method. Similarly, Laureanti et al. [38] reported that encapsulating pink pepper extract and green propolis extract via the freeze-drying method using a combination of GA and MD achieved higher encapsulation efficiency than using MD alone (98.3 vs. 93.3%).

Supporting our findings that encapsulation efficiency was higher with the 0MD:100GA formulation, Velazquez-Martinez et al. [61] reported that the use of GA as a wall material for encapsulation of bioactive compounds in sugarcane bagasse using the freeze-drying method resulted in a higher encapsulation efficiency than the use of MD alone (83% vs. 40%). Similarly, the freeze-drying encapsulation of a fennel oleoresin product using GA resulted in higher encapsulation efficiency than a blend of GA and MD (86.4 vs. 52.3%) [62]. This difference is primarily attributed to the structural properties of the encapsulated powders. The encapsulated powders developed using MD have a fragmented and incomplete structure, which may be associated with a lower retention capacity of bioactives. In contrast, the encapsulates prepared with GA are usually predominantly spherical in shape with minimal dents, an indication of a more cohesive and well-defined structure [63]. In addition, the difference in the molecular weight of GA and MD may cause a difference in the pore size distribution and morphology of the freeze-dried encapsulates, thereby affecting the encapsulation efficiency [61]. The lower emulsifying properties and surface activity of MD than GA [61] may also help to partially explain the higher encapsulation efficiency observed in 0MD:100GA formulation.

Storage temperature (room vs. cold temperature) did not affect eugenol concentrations over the 40-day storage (*p* = 0.43). As the storage duration progressed, the loss of eugenol increased, the extent of which was slightly greater in encapsulated powders developed only with MD than those with a blend of MD and GA (i.e., 50MD:50GA).

As shown in Table 6, the loss of the antioxidant capacity was slightly greater in the encapsulates stored at room temperature vs. those stored at cold temperature (8.14 vs. 5.90%). After 40 days of storage, the reduction in the antioxidant capacity was evident across all encapsulates, regardless of their wall material composition. However, the extent of this loss was slightly different. For example, after a 40-day storage at room temperature, the formulation containing 100% MD (100MD:0GA) experienced a greater loss of initial antioxidant activity compared to the formulation composed entirely of GA (0MD:100GA), with losses of 10.4% and 4.1%, respectively. This difference can be attributed to the high surface porosity of MD, which may facilitate the oxidation of active substances with potential antioxidant activity [7].

**Figure 2:**
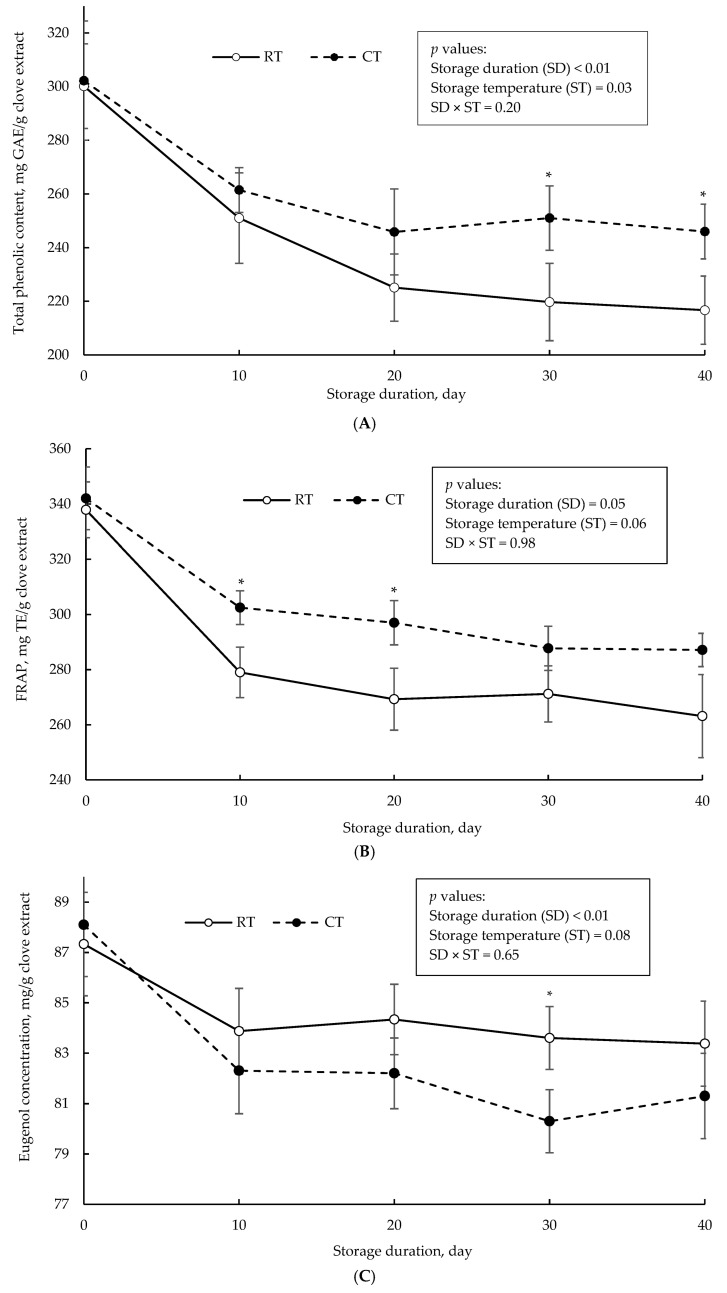
Total phenolic content (**A**), antioxidant capacity measured by FRAP assay (**B**), and eugenol concentration (**C**) of non-encapsulated clove extract over storage time at room temperature (RT; solid line) and cold temperature (CT; dotted line). Asterisks (*) indicate a significant difference (*p* < 0.05; Tukey’s test).

Total phenolics, antioxidant activity (measured using FRAP assay), and eugenol concentration of clove extract in the non-encapsulated form as a function of storage duration at room and cold temperature are illustrated in Figure 2. After 40-day storage, there was 27.8 and 18.6% loss in initial total phenolic content in clove extract stored under room temperature and cold temperature, respectively. Similarly, there was 22.1 and 16.1% loss in the antioxidant capacity of the clove extracts stored under room temperature and cold temperature, respectively. As the storage duration prolonged, the eugenol concentration began to decrease, with an average loss of 6.12% in the initial concentration after 40 days of storage in both room and cold temperature. Storage temperature had a slight effect (*p* = 0.08) on loss of initial eugenol during the 40-day storage (Figure 2C).

The trend in total phenolics and antioxidant capacity is consistent with a reported positive association between the phenolic content and antioxidant activity [64]. The magnitude of loss in the bioactivity of the clove extract (non-encapsulated form), as compared to the encapsulated form, may highlight the importance of the coating agent in protecting clove polyphenols and retaining the antioxidant capacity over an extended storage duration. This protective function is important in commercial settings to ensure the final product delivers consistent health benefits and optimal performance as the storage duration extends. The wall material typically functions as a physical barrier, decreasing the impact of deteriorative agents such as oxygen, heat, and light on the encapsulated powders [65]. Consistent with these findings, George et al. [15] reported that a 28-day storage of Moringa oleifera leaf extract in non-encapsulated form at 25 °C resulted in 27.1% loss in total phenolic content, while the encapsulation of the extract with a blend of MD and GA resulted in only an 11.1% loss in the initial total phenolic concentration. da Silva Júnior et al. [66] also observed a negligible impact of storage temperature (25 °C vs. 7 °C) on the total phenolic content of freeze-dried encapsulates of ciriguela peel extract in a 90-day storage stability test, which utilized MD and GA as wall materials.

Encapsulation using the freeze-drying method has been reported to result in products with irregular and porous structures [67], which is associated with the heightened susceptibility of phenolic compounds to oxidation degradation as compared to the spray-drying method. However, we did not identify a significant loss of phenolic compounds and eugenol in freeze-dried encapsulates (after 40-day storage), irrespective of their carrier agents. A longer-term evaluation of storage stability may reveal differences in the protective function according to the choice of carrier agents.

### 3.7. Accelerated Storage Stability

**Figure 3:**
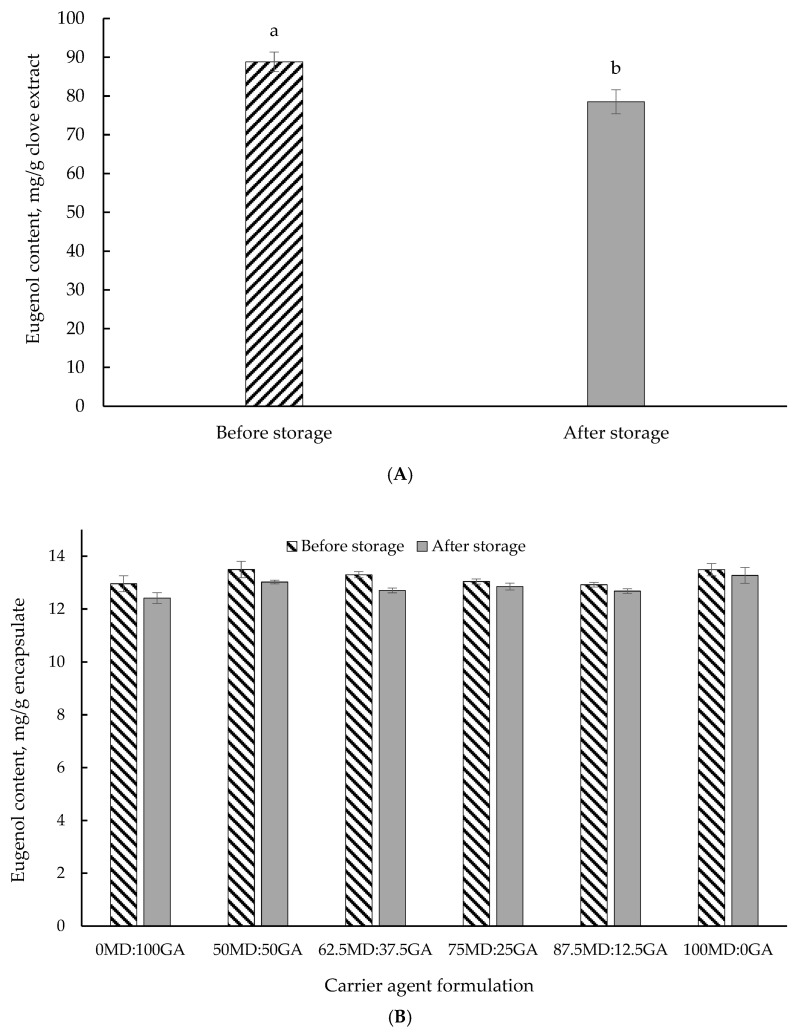
Eugenol concentration in clove extract in non-encapsulated form (**A**) and encapsulated form (**B**) before and after a 7-day accelerated storage stability test at 60 °C. MD = maltodextrin, GA = gum Arabic. Proportion of MD and GA in the formulations is presented in Table 1. Different letters (^a,b^) indicate a significant difference (*p* < 0.05; Tukey’s test).

Table 7 reports the changes in the total phenolic content of clove extract in the non-encapsulated and encapsulated forms using different wall material combinations after a 7-day accelerated storage stability test at 60 °C. Figure 3 also illustrates the eugenol concentration of clove extract in non-encapsulated (Figure 3A) or encapsulated form with different wall material combinations (Figure 3B) as a function of storage duration (7 days) at 60 °C. After 7 days of storage at 60 °C, the non-encapsulated clove extract experienced an 18.2% loss of total phenolics, resulting in a 27% loss of antioxidant capacity. Consistent with this observation, in a forced storage stability assay, Todorović et al. [68] reported a 29% reduction in total antioxidant capacity of bilberry extract stored at 60 °C for 5 days.

Generally, an increase in the total phenolic content was observed in encapsulates with a higher proportion of GA. This is likely owing to the elevated temperature impact promoting the hydrolysis of conjugated polyphenols that may potentially have led to the release of more free phenolic compounds [69]. These free phenolics may exhibit greater reactivity with the Folin–Ciocalteu reagent, resulting in a higher measured value of total phenolic content. In support of these findings, Robert et al. [70] reported a substantial increase (more than two-fold) in total phenolic content (measured using Folin–Ciocalteu reagent) of cactus pear pulp encapsulated with a combination of soybean protein isolate, inulin, or MD, after 35 days of storage at 60 °C. Overall, the findings of the accelerated storage stability test highlighted the protective effects of the wall materials, as demonstrated by slight losses in total phenolics and eugenol. One of the primary purposes of encapsulation is to protect the bioactive components against oxidation and deterioration, thereby minimizing their vulnerability to external factors that may compromise product quality and bioactivity. Our findings support the efficacy of encapsulation in fulfilling this important protective function under harsh conditions.

### 3.8. Phenolic Bioaccessibility

**Figure 4:**
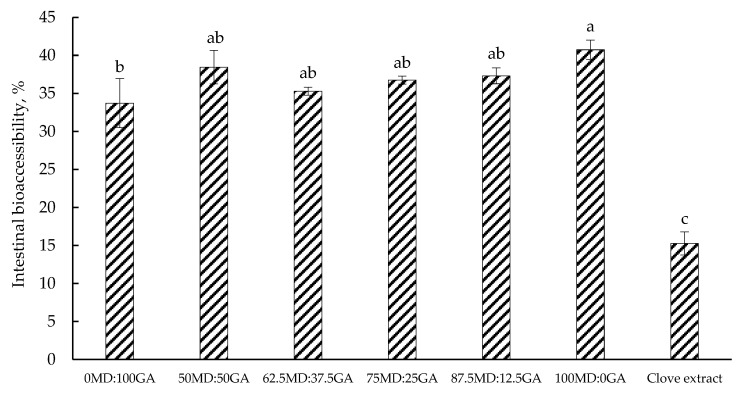
Intestinal bioaccessibility of total phenolics in clove extract in non-encapsulated or encapsulated form after simulated *in vitro* gastrointestinal digestion. MD = maltodextrin, GA = gum Arabic. Proportion of MD and GA in the formulations is presented in Table 1. Clove extract was freeze-dried powder in non-encapsulated form. Different letters (^a–c^) indicate significant differences (*p* < 0.05; Tukey’s test).

Figure 4 illustrates the bioaccessibility of total phenolics in clove extract, both in its non-encapsulated and encapsulated form, after intestinal digestion. Phenolic bioaccessibility was generally greater in encapsulated powders than clove extract without encapsulation. This may suggest that the encapsulation effectively protected the phenolic compounds throughout the *in vitro* gastrointestinal digestion. Similarly, in an *in vitro*-simulated gastrointestinal digestion, Silva et al. [71] reported a greater bioaccessibility of polyphenol-rich extract of green tea in non-encapsulated form vs. encapsulated using MD and cashew gum (24.2 vs. 28.2%). Ștefănescu et al. [72] investigated the phenolic bioaccessibility of Vaccinium leaf extracts in both encapsulated (80% MD + 20% glucose) and non-encapsulated forms through an *in vitro*-simulated gastrointestinal digestion and reported a similar trend across the species (e.g., 26.7 vs. 32.8% in case of bilberry extract). The decreased bioaccessibility, particularly in non-encapsulated clove extract, has been ascribed to the instability of phenolic compounds in the alkaline environment and their susceptibility to degradation by enzymes in the intestine [73]. Limited information is available in the literature regarding the bioaccessibility of clove extract polyphenols, which makes direct comparisons difficult for this experiment. More recently, Ozkan et al. [74] investigated the bioaccessibility of polyphenols in different herbs and reported a wide variation in their bioaccessibility, ranging from 16 to 61%, likely because of the differences in chemical structure and water solubility of the phenolic compounds in these herbs [27]. Gutiérrez-Grijalva et al. [75] also highlighted a substantial interspecies variation in oregano, with polyphenol bioaccessibility ranging from 5.75% to 90.4% across three different species.

Overall, the greater phenolic bioaccessibility of the encapsulated powders is likely because of the protective action of the carrier matrices on the bioactive compounds, as one of the main functions of encapsulation is to protect these compounds from degradation in the harsh environment of the gastric phase before they reach their targeted site in the intestine.

## 4. Conclusions

Our findings demonstrated that the freeze-drying encapsulation of clove extract using varying proportions of GA and MD produced encapsulated powders with different techno-functional properties. Although encapsulation using GA resulted in greater encapsulation efficiency, the physical properties were superior in MD encapsulates (i.e., moisture content, solubility, hygroscopicity, and bulk and tapped density). The encapsulation of the clove extract prevented a significant loss in the bioactive component and antioxidant capacity during storage under room and cold temperatures (up to 40 days) and even under extreme conditions (i.e., high temperature), necessary to enable the optimal delivery of health benefits. The encapsulation of the clove extract increased the bioaccessibility of clove polyphenols at the intestinal level as compared to non-encapsulated clove extract. Overall, our data suggest the efficacy of the encapsulation process in decreasing the bioactivity loss of the polyphenol-rich clove extract and improving storage stability and bioaccessibility. This positions encapsulation of clove extract as a promising approach in functional food applications. Future research should focus on further optimization of encapsulation formulations by investigating alternative encapsulating agents for improved techno-functional properties of the encapsulated clove extract. Our future animal model trial using chicken aims to evaluate the efficacy of feeding encapsulated clove extract in ameliorating heat-stress-induced impairments and promoting gut health.

## Figures and Tables

**Table 1:** Proportions of maltodextrin (MD) and gum Arabic (GA) in encapsulation formulations.

Formulations	Maltodextrin, %	Gum Arabic, %
0MD:100GA	0	100
50MD:50GA	50	50
62.5MD:37.5GA	62.5	37.5
75MD:25GA	75	25
87.5MD:12.5GA	87.5	12.5
100MD:0GA	100	0

**Table 2:** Physicochemical properties and flowability characteristics of clove extract encapsulated with different wall materials.

Formulations *	Moisture, %	Solubility, %	WAC, g/g	Water Activity	Hygroscopicity, %	ρ_bulk_, g/cm^3^	ρ_tapped_, g/cm^3^	HR	CI, %
0MD:100GA	5.30 ± 0.33 ^a^	89.9 ± 0.76 ^b^	0.34 ± 0.08	0.29 ± 0.02	10.1 ± 0.40 ^a^	0.310 ± 0.01 ^b^	0.409 ± 0.01 ^b^	1.32 ± 0.01	24.3 ± 0.65
50MD:50GA	3.24 ± 0.76 ^b^	90.0 ± 1.14 ^b^	0.34 ± 0.07	0.26 ± 0.03	9.10 ± 0.26 ^a^	0.314 ± 0.02 ^b^	0.406 ± 0.01 ^b^	1.29 ± 0.02	22.6 ± 1.01
62.5MD:37.5GA	2.44 ± 0.09 ^bc^	91.8 ± 0.73 ^ab^	0.35 ± 0.08	0.24 ± 0.03	7.17 ± 0.36 ^b^	0.307 ± 0.01 ^b^	0.408 ± 0.01 ^b^	1.33 ± 0.05	24.7 ± 2.85
75MD:25GA	2.98 ± 0.58 ^b^	92.5 ± 0.81 ^a^	0.33 ± 0.07	0.23 ± 0.01	6.67 ± 0.47 ^b^	0.309 ± 0.02 ^b^	0.408 ± 0.01 ^b^	1.32 ± 0.03	24.4 ± 1.57
87.5MD:12.5GA	2.89 ± 0.71 ^b^	92.7 ± 0.61 ^a^	0.34 ± 0.06	0.28 ± 0.02	6.74 ± 0.45 ^b^	0.325 ± 0.01 ^ab^	0.429 ± 0.01 ^ab^	1.32 ± 0.04	24.3 ± 2.29
100MD:0GA	1.39 ± 0.26 ^c^	93.3 ± 0.82 ^a^	0.32 ± 0.08	0.28 ± 0.03	6.70 ± 0.36 ^b^	0.353 ± 0.01 ^a^	0.439 ± 0.01 ^a^	1.25 ± 0.03	19.7 ± 2.16
*p* value	<0.01	<0.01	0.96	0.17	<0.01	<0.01	<0.01	0.15	0.14

* MD = maltodextrin, GA = gum Arabic. Proportion of MD and GA in the formulations is presented in Table 1. WAC = water absorption capacity. ρ_bulk_ = bulk density. ρ_tapped_ = tapped density. HR = Hausner’s ratio. CI = Carr index ^a–c^ Means with different superscripts within a column differ (Tukey’s test).

**Table 3:** Colorimetric analysis of clove extract in non-encapsulated or encapsulated form with different wall materials.

Formulations *	*L*	*a*	*b*
0MD:100GA	53.1 ± 2.10 ^b^	11.2 ± 0.33 ^b^	30.5 ± 0.85
50MD:50GA	57.7 ± 1.93 ^a^	11.3 ± 0.37 ^b^	30.9 ± 0.94
62.5MD:37.5GA	57.5 ± 3.07 ^a^	11.4 ± 0.25 ^b^	31.3 ± 1.06
75MD:25GA	57.4 ± 2.94 ^a^	11.3 ± 0.39 ^b^	31.0 ± 1.12
87.5MD:12.5GA	59.0 ± 3.08 ^a^	11.0 ± 0.31 ^b^	30.2 ± 0.92
100MD:0GA	59.3 ± 2.69 ^a^	11.1 ± 0.39 ^b^	31.1 ± 0.77
Clove extract ^1^	45.4 ± 2.94 ^c^	14.8 ± 0.99 ^a^	31.2 ± 1.88
*p* value	<0.01	<0.01	0.63

* MD = maltodextrin, GA = gum Arabic. Proportion of MD and GA in the formulations is presented in Table 1. ^1^ Freeze-dried powder in non-encapsulated form. *L* represents brightness, ranging from 0 (darkness) to 100 (lightness). *a* represents color shift from greenness (negative) to redness (positive). *b* represents color shift from blueness (negative) to yellowness (positive). ^a–c^ Means with different superscripts within a column differ (Tukey’s test).

**Table 4:** Solid recovery and loading efficiency of total phenolics and eugenol in clove extract encapsulated with different wall materials.

Formulations *	Solid Recovery, %	Total Phenolics, %	Eugenol, %
0MD:100GA	93.8 ± 1.30	78.5 ± 3.18	74.2 ± 1.79
50MD:50GA	93.2 ± 2.03	80.1 ± 2.53	73.2 ± 2.14
62.5MD:37.5GA	92.0 ± 2.01	79.1 ± 2.83	75.6 ± 0.95
75MD:25GA	93.4 ± 1.32	81.6 ± 2.56	74.8 ± 0.71
87.5MD:12.5GA	93.9 ± 0.92	79.0 ± 3.55	72.4 ± 1.11
100MD:0GA	92.6 ± 1.92	79.5 ± 1.75	70.5 ± 2.06
*p* value	0.84	0.69	0.78

* MD = maltodextrin, GA = gum Arabic. Proportion of MD and GA in the formulations is presented in Table 1.

**Table 5:** Polydispersity index (PDI) and zeta potential of clove extract encapsulated with different wall materials.

Formulations *	PDI	Zeta Potential (mV)
0MD:100GA	0.57 ± 0.10 ^a^	−23.2 ± 2.67 ^b^
50MD:50GA	0.52 ± 0.07 ^ab^	−22.1 ± 1.89 ^b^
62.5MD:37.5GA	0.55 ± 0.05 ^ab^	−22.4 ± 0.77 ^b^
75MD:25GA	0.59 ± 0.12 ^ab^	−25.3 ± 1.23 ^ab^
87.5MD:12.5GA	0.47 ± 0.11 ^b^	−22.9 ± 0.68 ^b^
100MD:0GA	0.68 ± 0.11 ^a^	−29.7 ± 2.27 ^a^
Clove extract ^1^	0.52 ± 0.11 ^a^	−28.6 ± 0.71 ^a^
*p* value	0.02	<0.01

* MD = maltodextrin, GA = gum Arabic. Proportion of MD and GA in the formulations is presented in Table 1. ^1^ Freeze-dried powder in non-encapsulated form. ^a,b^ Means with different superscripts within a column differ (Tukey’s test).

**Table 6:** Surface and core phenolic contents, encapsulation efficiency, eugenol concentration, and antioxidant capacity (measured as FRAP) as influenced by encapsulation formulations, storage temperature, and storage duration *.

Items	Room Temperature					Cold Temperature					***p* Value**			
	Storage Duration, Day					Storage Duration, Day								
	0	10	20	30	40	0	10	20	30	40	**Trt.**	**ST**	**SD**	**ST × SD**
Surface phenolics, mg GAE/g											<0.01	<0.01	0.01	0.39
0MD:100GA	2.03 ± 0.25	2.07 ± 0.10	2.09 ± 0.20	2.25 ± 0.17	2.61 ± 0.42	1.93 ± 0.35	1.91 ± 0.21	2.04 ± 0.29	2.18 ± 0.34	2.34 ± 0.27				
50MD:50GA	2.77 ± 0.34	2.71 ± 0.23	2.85 ± 0.29	2.77 ± 0.46	2.95 ± 0.32	2.73 ± 035	2.90 ± 0.43	2.82 ± 0.38	2.90 ± 0.36	3.02 ± 0.17				
62.5MD:37.5GA	3.10 ± 0.26	3.22 ± 0.38	3.13 ± 0.30	3.29 ± 0.36	4.18 ± 0.96	3.13 ± 0.31	3.17 ± 0.31	3.16 ± 0.25	3.69 ± 0.22	3.54 ± 0.31				
75MD:25GA	3.27 ± 0.21	3.35 ± 0.17	3.62 ± 0.23	4.03 ± 0.17	4.15 ± 0.41	3.17 ± 0.32	3.03 ± 0.18	3.05 ± 0.37	3.18 ± 0.38	3.40 ± 0.24				
87.5MD:12.5GA	3.10 ± 0.27	2.92 ± 0.18	3.12 ± 0.29	3.72 ± 0.28	3.73 ± 0.29	3.04 ± 0.26	2.99 ± 0.21	3.14 ± 0.24	3.41 ± 0.37	3.35 ± 0.25				
100MD:0GA	3.20 ± 0.20	3.22 ± 0.32	3.40 ± 0.21	3.67 ± 0.56	3.71 ± 0.23	3.09 ± 0.28	3.03 ± 0.28	3.13 ± 0.28	3.16 ± 0.35	3.22 ± 0.30				
Core phenolics, mg GAE/g											0.26	0.04	0.01	0.34
0MD:100GA	39.6 ± 1.92	40.5 ± 1.14	39.2 ± 0.78	36.7 ± 2.49	37.5 ± 1.89	38.7 ± 0.53	40.7 ± 2.14	41.5 ± 1.75	35.9 ± 1.87	36.0 ± 1.73				
50MD:50GA	39.6 ± 2.15	40.1 ± 2.50	38.9 ± 1.21	39.0 ± 1.57	39.8 ± 2.71	39.3 ± 1.97	40.8 ± 2.22	40.4 ± 3.10	38.0 ± 2.61	38.3 ± 1.66				
62.5MD:37.5GA	39.5 ± 3.42	40.2 ± 1.34	40.1 ± 2.00	40.6 ± 1.86	37.0 ± 1.31	38.7 ± 1.53	40.3 ± 2.55	39.2 ± 2.05	38.2 ± 1.66	38.5 ± 1.90				
75MD:25GA	40.1 ± 2.12	40.9 ± 2.82	38.5 ± 1.53	38.0 ± 1.32	39.5 ± 2.18	40.1 ± 2.10	40.5 ± 2.48	42.8 ± 3.44	39.1 ± 1.15	38.8 ± 2.70				
87.5MD:12.5GA	38.5 ± 1.98	39.8 ± 2.29	40.1 ± 2.05	38.4 ± 1.66	41.3 ± 2.04	38.1 ± 2.58	37.3 ± 1.80	37.2 ± 2.25	37.4 ± 2.45	37.6 ± 1.63				
100MD:0GA	37.4 ± 2.21	40.0 ± 2.05	39.1 ± 1.30	41.8 ± 3.92	40.8 ± 4.32	37.7 ± 2.72	39.4 ± 2.57	38.1 ± 2.41	37.7 ± 2.77	37.0 ± 2.03				
Encapsulation efficiency, %											<0.01	0.12	<0.01	0.77
0MD:100GA	94.9 ± 0.69	94.9 ± 0.35	94.7 ± 0.51	93.9 ± 0.13	93.0 ± 0.61	95.0 ± 0.95	95.3 ± 0.68	95.1 ± 0.64	93.9 ± 1.08	93.5 ± 0.87				
50MD:50GA	93.0 ± 0.95	93.2 ± 0.89	92.7 ± 0.74	92.9 ± 1.01	92.6 ± 0.40	93.1 ± 0.55	92.9 ± 0.78	93.0 ± 1.27	92.4 ± 1.12	92.1 ± 0.15				
62.5MD:37.5GA	92.1 ± 1.23	92.1 ± 1.04	92.2 ± 1.15	91.9 ± 0.81	88.7 ± 2.38	91.9 ± 0.57	92.1 ± 0.76	91.9 ± 1.10	90.3 ± 0.55	90.8 ± 1.20				
75MD:25GA	91.9 ± 0.89	91.8 ± 0.78	90.6 ± 0.95	89.4 ± 0.42	89.4 ± 1.65	92.1 ± 0.76	92.5 ± 0.25	92.9 ± 0.76	91.9 ± 0.78	91.3 ± 0.16				
87.5MD:12.5GA	92.0 ± 1.15	92.7 ± 0.40	92.2 ± 0.31	90.3 ± 0.31	90.9 ± 0.87	92.0 ± 0.18	92.0 ± 0.81	91.6 ± 1.07	90.9 ± 0.64	91.1 ± 0.26				
100MD:0GA	91.4 ± 0.84	92.0 ± 0.57	91.3 ± 0.36	91.2 ± 0.70	90.9 ± 0.44	91.8 ± 1.40	92.2 ± 1.18	91.8 ± 0.55	91.6 ± 0.35	91.3 ± 0.36				
Eugenol, mg/g											<0.01	0.43	<0.01	0.98
0MD:100GA	12.8 ± 0.45	12.0 ± 0.56	12.1 ± 0.47	12.1 ± 0.50	11.7 ± 0.59	12.8 ± 0.28	12.6 ± 0.65	12.4 ± 0.29	12.1 ± 0.24	11.5 ± 0.37				
50MD:50GA	12.6 ± 0.62	12.3 ± 0.37	12.3 ± 0.49	12.2 ± 0.41	12.3 ± 0.35	12.7 ± 0.21	12.3 ± 0.24	12.3 ± 0.53	12.1 ± 0.38	12.1 ± 0.25				
62.5MD:37.5GA	13.2 ± 0.53	13.0 ± 0.25	13.1 ± 0.32	12.8 ± 0.40	12.5 ± 0.49	13.0 ± 0.71	13.1 ± 0.73	12.9 ± 0.21	12.8 ± 0.29	12.6 ± 0.43				
75MD:25GA	13.1 ± 0.24	12.8 ± 0.61	12.9 ± 0.49	12.5 ± 0.49	12.2 ± 0.46	12.8 ± 0.49	12.4 ± 0.62	12.5 ± 0.41	12.1 ± 0.34	12.4 ± 0.32				
87.5MD:12.5GA	12.9 ± 0.41	12.7 ± 0.27	12.8 ± 0.36	11.8 ± 0.77	11.8 ± 0.11	13.1 ± 0.25	12.7 ± 0.29	12.4 ± 0.82	12.3 ± 0.57	12.1 ± 0.41				
100MD:0GA	12.8 ± 0.86	12.2 ± 0.50	11.5 ± 0.38	11.2 ± 0.41	11.3 ± 0.28	12.6 ± 0.47	12.1 ± 0.21	11.8 ± 0.34	11.9 ± 0.53	11.6 ± 0.39				
FRAP, mg TE/g											0.02	<0.01	<0.01	0.69
0MD:100GA	74.9 ± 1.62	70.1 ± 1.98	70.2 ± 2.96	70.8 ± 2.25	71.5 ± 4.94	77.2 ± 3.72	76.7 ± 1.75	75.3 ± 2.11	72.9 ± 3.13	71.5 ± 1.20				
50MD:50GA	76.4 ± 2.16	73.6 ± 0.54	74.0 ± 1.97	72.7 ± 0.85	72.4 ± 0.90	77.2 ± 1.90	74.9 ± 2.25	73.6 ± 2.11	70.3 ± 5.11	71.5 ± 1.81				
62.5MD:37.5GA	76.9 ± 1.30	75.2 ± 1.56	72.1 ± 3.59	70.5 ± 3.04	70.8 ± 3.66	77.1 ± 1.36	76.7 ± 2.70	75.5 ± 0.72	73.2 ± 2.69	72.9 ± 4.16				
75MD:25GA	77.0 ± 1.27	77.0 ± 1.29	75.9 ± 2.11	71.4 ± 3.64	70.1 ± 4.09	76.3 ± 1.91	75.2 ± 1.50	72.8 ± 2.70	70.6 ± 3.87	71.0 ± 2.79				
87.5MD:12.5GA	75.9 ± 1.35	72.7 ± 1.65	69.9 ± 3.67	70.4 ± 3.46	67.8 ± 1.28	75.5 ± 2.06	73.7 ± 1.98	72.2 ± 4.68	74.2 ± 1.24	71.7 ± 2.81				
100MD:0GA	75.0 ± 0.70	71.1 ± 1.19	69.8 ± 2.94	68.5 ± 3.60	66.2 ± 1.47	76.0 ± 1.83	73.7 ± 4.72	73.4 ± 2.95	74.2 ± 1.75	73.4 ± 1.34				

* Values are mean of three replications ± standard deviation. MD = maltodextrin, GA = gum Arabic. Proportion of MD and GA in the formulations is presented in Table 1. Trt. = treatment effect. ST = storage temperature. SD = storage duration effect. ST × SD = interaction. FRAP = ferric reducing antioxidant power.

**Table 7:** Total phenolic content of clove extract in non-encapsulated form and encapsulated form using different wall material combinations before and after a 7-day accelerated storage stability test at 60 °C.

Formulations *	Total Phenolic Content, mg GAE/g	
	Before	After
0MD:100GA	40.3 ± 0.75	51.4 ± 0.56
50MD:50GA	40.5 ± 0.53	44.0 ± 0.41
62.5MD:37.5GA	41.4 ± 1.35	45.8 ± 0.72
75MD:25GA	41.3 ± 0.36	44.2 ± 0.90
87.5MD:12.5GA	41.8 ± 0.29	43.6 ± 1.61
100MD:0GA	41.2 ± 1.15	41.0 ± 0.59
Clove extract ^1^	308 ± 11.6	253 ± 7.74

* MD = maltodextrin, GA = gum Arabic. Proportion of MD and GA in the formulations is presented in Table 1. ^1^ Freeze-dried powder in non-encapsulated form.

## Data Availability

The original contributions presented in this study are included in the article/Supplementary Material. Further inquiries can be directed to the corresponding author.

## Supplementary Materials

The following supporting information can be downloaded at: https://www.mdpi.com/article/10.3390/foods14020237/s1, Supplementary Figure S1. Calibration curve data for the HPLC analysis of eugenol in experimental samples.
