# Supplementary material for: Physicochemical Characterization, Storage Stability Behavior, and Intestinal Bioaccessibility of Clove Extract Encapsulated Using Varying Combinations of Gum Arabic and Maltodextrin
Source: Foods. 2025 Jan 14;14(2):237. doi: 10.3390/foods14020237 (PMC11764740; doi:10.3390/foods14020237)
Supplement: Supplementary file 1 [file foods-14-00237-s001.zip › foods-3395248-supplementary.pdf]

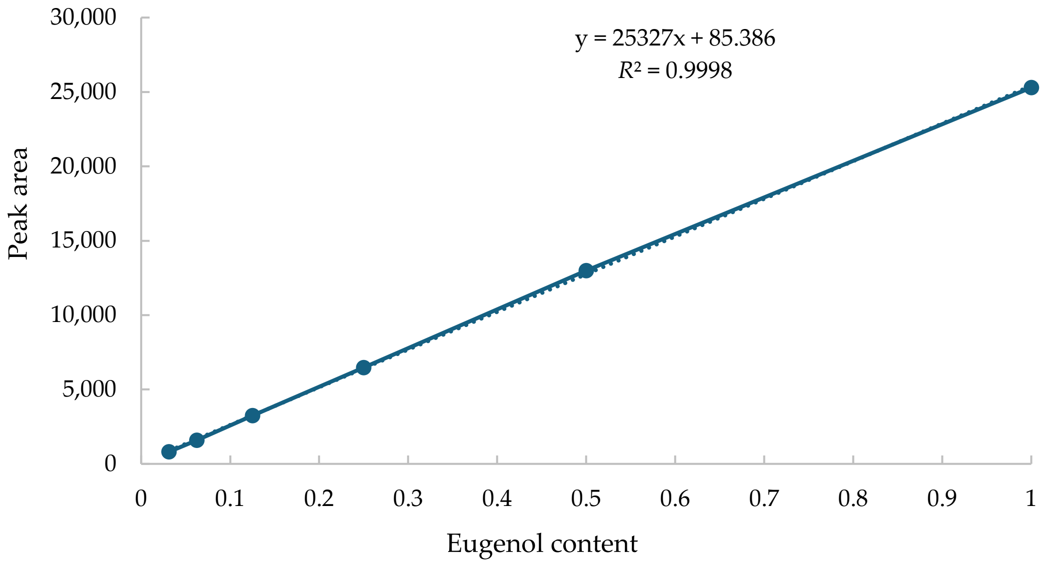

Supplementary Figure S1. Calibration curve data for the HPLC analysis of eugenol in experimental samples. A linear relationship was observed between eugenol concentration and peak area (R^2 = 0.9998).
